# On the Color of the Hair

**Published:** 1849-08

**Authors:** 


*On the Color of the Hair.*—In the Medical Gazette for November 17, 1848, Dr. Griffith states that the appearance of pigment-cells in the hair, as seen under the microscope, is deceptive; and that this appearance results from a number of air cavities existing in the medullary portion of the hair. The air refracts the rays of light beyond the field of the microscope, and they appear black, with generally, however, a white spot in the centre. He considers his statement proved by the following observations:—

1. If a piece be cut, transversely, from the centre of the hair,* and this be digested in warm water or alcohol, the hair becomes very transparent; and, by this means, all the air-cavities may be filled with the water or spirit,—nay, if the piece of hair be immersed in oil of turpentine, and warmed, the fluid may be seen under the microscope to enter the cells, and the air to escape in bubbles at the ends. All appearance of the pigment then vanishes; but traces of the cell-wall of the medulla are still faintly seen,—they not being of the same refractive power as the medium in which they are immersed.

2. If the portion of hair be removed from the water, spirit, or oil, and allowed to dry, the fluid evaporates, and the air may be seen under the microscope to enter and restore the original appearance. On preserving specimens of hair in Canada balsam, the cells are frequently filled, in parts, with the balsam, especially at the extremities.

3. If the hair be bruised in an agate mortar, it becomes flattened out, resembling a shred of membrane, the pigment appearance being completely destroyed.—*Ibid.*

* That of the sable, or some other animal in which the cavities are large and distinct, is the best.

